# 

**Published:** 1889-01

**Authors:** 


COLLABORATORS:

CHICAGO:

Professor E. ANDREWS, M.D., LL.D.
Professor J. BARTLETT, M.D.
Professor W. T. BELFIELD, M.D.
Professor F. BILLINGS, M.D.
Professor W. H. BYFORD, A.M., M.D.
Professor L. CURTIS, A.M., M.D.
Professor N. S. DAVIS, Jr., A.M., M.D.
Professor O. C. DE WOLF, A.M., M.D.
Professor C. FENGER, M.D.
Professor J. N. HYDE, A.M., M.D.
Professor E. F. INGALS, A.M., M.D.
Professor F. S. JOHNSON, A.M., M.D.
Professor H. A. JOHNSON, M.D., LL.D.
Professor C. W. PURDY, M.D.
Professor C. G. SMITH, A.M., M.D.
Professor E. WING, A.M., M.D.
Professor E. C. DUDLEY, A.B., M.D.

MILWAUKEE, WIS.:
Professor N. SENN, M.D., Ph.D.

WASHINGTON, D. C.:

S. O. RICHEY, M. D.

UNITED STATES ARMY:
Surgeon D. L. HUNTINGTON, M.D.

UNITED STATES NAVY:
Surgeon-General J. M. BROWNE, M.D. Medical-Director A. L. GIHON, A.M., M.D.

MARINE HOSPITAL SERVICE:
Surgeon S. T. ARMSTRONG, M.D., Ph.D.

